# Depression Associated With Hormonal Contraceptive Use as a Risk Indicator for Postpartum Depression

**DOI:** 10.1001/jamapsychiatry.2023.0807

**Published:** 2023-04-26

**Authors:** Søren Vinther Larsen, Anders Pretzmann Mikkelsen, Øjvind Lidegaard, Vibe Gedso Frokjaer

**Affiliations:** 1 Neurobiology Research Unit, Copenhagen University Hospital, Rigshospitalet, Copenhagen, Denmark; 2 Department of Clinical Medicine, University of Copenhagen, Copenhagen, Denmark; 3 Department of Gynaecology and Obstetrics, Juliane Marie Centre, Copenhagen University Hospital, Rigshospitalet, Copenhagen, Denmark; 4 Department of Gynaecology and Obstetrics, Copenhagen University Hospital, Herlev and Gentofte Hospital, Herlev, Denmark; 5 Psychiatric Center Copenhagen, Mental Health Services in the Capital Region of Denmark, Copenhagen, Denmark

## Abstract

**Importance:**

Hormonal sensitivity may contribute to the risk of depression in some women, as observed during the premenstrual, postpartum, and perimenopausal phases, and when initiating hormonal contraception (HC). However, little evidence exists to support that such depressive episodes are linked across the reproductive life span.

**Objective:**

To determine whether prior depression associated with HC initiation is coupled with a higher risk of postpartum depression (PPD) than prior depression not associated with HC initiation.

**Design, Setting, and Participants:**

This cohort study used Danish health registry data collected from January 1, 1995, through December 31, 2017, and analyzed from March 1, 2021, through January 1, 2023. All women living in Denmark born after 1978 with their first delivery between January 1, 1996, and June 30, 2017, were eligible for inclusion; 269 354 met these criteria. Women were then excluded if they had never used HC or if they had a depressive episode before 1996 or within 12 months prior to delivery.

**Exposures:**

Prior depression associated with vs not associated with HC initiation, ie, if developed within 6 months after start of an HC exposure or not. Depression was defined as a hospital diagnosis of depression or filling a prescription for antidepressant medication.

**Main Outcomes and Measures:**

Crude and adjusted odds ratios (ORs) were calculated for the incidence of PPD defined as the development of depression within 6 months after first delivery.

**Results:**

Of 188 648 first-time mothers, 5722 (3.0%) (mean [SD] age, 26.7 [3.9] years) had a history of depression associated with initiation of HC use, and 18 431 (9.8%) (mean [SD] age, 27.1 [3.8] years) had a history of depression not associated with the initiation of HC. Women with HC-associated depression had a higher risk of PPD than women with prior non–HC-associated depression (crude OR, 1.42 [95% CI, 1.24-1.64]; adjusted OR, 1.35 [95% CI, 1.17-1.56]).

**Conclusions and Relevance:**

These findings suggest that a history of HC-associated depression may be associated with a higher risk of PPD, supporting that HC-associated depression may indicate PPD susceptibility. This finding offers a novel strategy in clinical PPD risk stratification and points to the existence of a hormone-sensitive subgroup of women.

## Key Points

**Question:**

Is prior hormonal contraception (HC)–associated depression associated with a higher risk of postpartum depression compared with prior depression not associated with HC use?

**Findings:**

In this cohort study of 188 648 first-time mothers, prior depression after initiation of HC was associated with a higher risk of postpartum depression than prior depression not associated with HC initiation.

**Meaning:**

The study’s findings suggest that depression associated with HC use can indicate postpartum depression susceptibility and may provide evidence for a link between depressive episodes with possible hormonal contributions and point to the existence of a subgroup of women sensitive to hormonal transitions across their reproductive life spans.

## Teaser

This cohort study investigates previous depression associated with hormonal contraceptive use and risk for postpartum depression in Danish first-time mothers.

## Introduction

Women are approximately twice as likely to develop depressive episodes compared with men.^1^ This gap between sexes starts during adolescence, which coincides with menarche in girls, and lasts until menopause.^2^ Hence, a woman’s reproductive life span is a time of heightened vulnerability for depression, aligning with an increased risk of depression associated with hormonal transitions across the menstrual cycle, when 3% to 8% of women experience premenstrual dysphoric disorder (PMDD)^3^; the peripartum period, when approximately 13% of women experience postpartum depression (PPD)^4^; and the perimenopausal period, when large estradiol fluctuations predict risk of perimenopausal depression.^5^ Concerningly, initiating hormonal contraception (HC) also has been associated with an increased risk of developing a depressive episode.^6,7^

Women experiencing depressive episodes associated with hormonal transitions may comprise a certain hormone-sensitive subgroup of women within the broader diagnostic category of major depressive disorder. Since treatment of major depressive disorder is far from optimal, identification of relevant subgroups with distinct etiologic contributions to the disorder and responsiveness to certain triggers or treatments would help to build a much-needed rationale for precision medicine in psychiatry. However, little is known about whether the depressive episodes across women’s reproductive lives share similar etiology or whether they are linked.^8^ Some evidence supports that women with PPD are more likely to have a history of PMDD and that women who experience depressive symptoms in perimenopause are more likely to have a history of PPD and PMDD.^9,10^ However, the evidence is based on retrospective reports susceptible to recall and confirmation biases and lack the use of confirmed clinical diagnoses and, therefore, represent only limited evidence. The few studies that have investigated depressive symptoms associated with hormonal transitions and HC-associated mood deterioration are likely underpowered and have shown inconsistent results.^11,12,13,14,15^ Therefore, large-scale observational studies spanning the reproductive age are needed to shed light on the complex associations between depressive episodes occurring throughout women’s lives.

This study takes advantage of Danish national health registers to evaluate the existence of a subgroup of women who are prone to develop depressive episodes across hormonal transitions, including transitions induced by exogenous hormone exposure in terms of HC. We examined whether such depressive episodes are associated with one another across a woman’s reproductive life span; specifically, we examined whether a history of a depressive episode associated with initiation of HC poses a higher risk for later PPD compared with a history of depression not associated with HC initiation.

## Methods

### Study Design

This population-based cohort study is based on health care data from Danish national registers. The specific registers and variables used are listed in eTable 1 in Supplement 1. Data were provided by the Danish eHealth Authority hosted by Statistics Denmark and linked via the unique personal identification number given to Danish residents at birth or immigration. Approval of the study was achieved through the Danish Data Protection Agency (journal No. Pactius-2020-217). According to Danish law, no ethics approval or informed consent are needed for register-based studies. The study followed the Strengthening the Reporting of Observational Studies in Epidemiology (STROBE) reporting guideline.^16^

### Study Population

The study population included all women in Denmark born after 1978 (ie, women aged a maximum of 16 years in 1995) who delivered their first child between January 1, 1996, and June 30, 2017, according to the Danish Civil Registration System and the Medical Birth Registry.^17,18^ Women were excluded if they (1) had never used HC (to minimize potential confounding associated with personality or behavior associated with HC use and depression susceptibility and to test for HC sensitivity before pregnancy); (2) immigrated at 16 years or older or emigrated for more than 6 consecutive months after turning 16; (3) had a depressive episode before 1996 or within 12 months prior to delivery, as this could indicate an ongoing depression while entering pregnancy; and (4) had a multiple birth or stillbirth.

### Exposures

The exposure of interest was prior depression associated with initiation of HC defined as a depressive episode that developed within 6 months after the start of HC exposure, as depression risk seems to peak within this period.^6^ Start of HC exposure was defined as the start or restart of HC use or a change in type of HC used (ie, when a change in the Anatomical Therapeutic Chemical code was registered). To ensure that a depressive episode could only be linked to a new HC exposure, a restart was registered if a new prescription happened more than 6 months after the end of the duration of the last prescription (the duration of implants and hormonal intrauterine device prescriptions was set to 1000 days). A depressive episode was defined as filling a prescription of antidepressant medication or obtaining a depression discharge diagnosis from an inpatient or outpatient psychiatric clinic with the admission day as the index date identified in the Psychiatric Central Register.^19^ To distinguish multiple depressive episodes, a new depressive episode was registered if 1 of the following criteria was met: (1) when a new prescription was filled later than the end of the duration of the last prescription plus a 30-day grace period^20^ or a minimum of 6 months after a depression discharge diagnosis or (2) when a depression discharge diagnosis occurred a minimum of 6 months after the end of a treatment period or after a previous depression discharge diagnosis. The duration of a prescription was calculated by multiplying the number of packages dispensed by the number of defined daily doses per package. If more prescriptions were dispensed on the same day, the duration was calculated as the sum of the treatment days for each prescription. In cases of multiple depressive episodes, having 1 episode associated with initiation of HC exposure was used to define a history of HC-associated depression. Women not fulfilling this definition were considered to have a history of non–HC-associated depression or no history of depression.

### Outcome and Covariates

The outcome was PPD, which was defined as filling a prescription for antidepressant medication or obtaining a hospital discharge diagnosis of depression within 6 months after first childbirth according to the National Prescription Register or National Patient Register, respectively.^21,22^ To adjust for potential confounders, we obtained information on maternal age at delivery (younger than 20 years and 5-year bands thereafter); highest educational level at delivery (less than high school, high school or vocational education, or bachelor’s degree or higher); family history of depression, defined as having a parent with a depression diagnosis; civil status (married or not); and potential obstetric risk factors, including preterm birth, instrument-assisted or cesarean delivery, preeclampsia or eclampsia, and pregestational or gestational diabetes. Furthermore, we acquired information on other potential confounding factors, such as other major psychiatric disorders, including organic mental disorders, mental and behavioral disorders due to substance use, schizophrenia, bipolar disorder, eating disorders, and mental disability; and medical indications for HC use, including polycystic ovary syndrome, endometriosis, premenstrual syndrome, dysmenorrhea, heavy menstrual bleeding, hirsutism, and acne. We also controlled for time trends in depression diagnostics and prescriptions of antidepressants by including calendar year in 5-year bands.

### Statistical Analysis

Analyses were conducted between March 1, 2021, and January 1, 2023. We used logistic regression to calculate odds ratios (ORs) among the 3 exposure groups: (1) history of non–HC-associated depression, (2) history of HC-associated depression, and (3) no history of depression. The first group was used as the reference. We calculated crude ORs and ORs adjusted for the listed covariates. Estimates were interpreted as relative risks according to the rare disease assumption.^23^

### Sensitivity Analyses

We conducted 5 sensitivity analyses. First, we removed obstetric risk factors from the adjustment set to minimize the risk of overadjusting the model. Second, we used perinatal depression as an outcome, ie, we included depressive episodes developed late in pregnancy (in the third trimester) and post partum to address that depressive episodes frequently emerge in late pregnancy.^24^ Third, we excluded mothers who started using HC after delivery but before they developed PPD. Fourth, we increased the grace period from 30 days to 90 and 180 days to distinguish a new prescription of antidepressant medication. Fifth, exposure classification was restricted to the first depressive episode, which was repeated with the framework from the first and second sensitivity analyses. Odds ratios were calculated with 95% CIs, and the null-hypothesis was rejected if they did not overlap 1.00. All analyses were conducted using R, version 4.1.3 statistical software (R Foundation for Statistical Computing).

## Results

**Figure 1.**
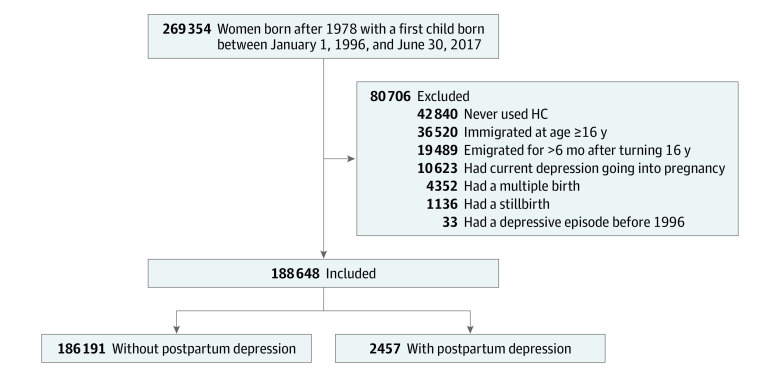
Study Population The study population was derived from the source population. The same individual could have met multiple exclusion criteria; thus, the sum of the numbers from all exclusion criteria does not equal the total number excluded. HC indicates hormonal contraception.

The study population included 188 648 first-time mothers (Figure 1). Of all 269 354 eligible women, 84% had used HC before their first child was born. Of the study population, 2457 developed PPD, corresponding to an incidence rate of 1.3%. Furthermore, 5722 first-time mothers (3.0%; mean [SD] age, 26.7 [3.9] years) had a history of HC-associated depression, 18 431 (9.8%; mean age [SD], 27.1 [3.8] years) had a history of non–HC-associated depression, and 164 495 (87.2%; mean age [SD], 26.3 [3.9] years) had no history of depression. A summary of demographic characteristics and clinical profiles among the exposure groups is shown in Table 1. Notably, women with HC-associated depression had more depressive episodes than women with non–HC-associated depression, with 63.4% vs 38.6% having had more than 1 episode, respectively.

**Table 1.** Demographic Characteristics and Clinical Profiles

Profile	Exposure, No. (%)		
	Non–HC-associated depression	HC-associated depression	No depression
Total	18 431 (9.8)	5722 (3.0)	164 495 (87.2)
Maternal age at delivery, y			
<20	415 (2.3)	191 (3.3)	9880 (6.0)
20-24	5221 (28.3)	1830 (32.0)	49 885 (30.3)
25-29	8656 (47.0)	2527 (44.2)	77 738 (47.3)
30-34	3721 (20.2)	1059 (18.5)	25 171 (15.3)
35-39	418 (2.3)	115 (2.0)	1821 (1.1)
Educational level			
Less than high school	5571 (30.2)	2143 (37.5)	37 035 (22.5)
High school or vocational education	7735 (42.0)	2174 (38.0)	66 645 (40.5)
Bachelor’s degree or higher	5125 (27.8)	1405 (24.6)	60 815 (37.0)
Married	8402 (45.6)	2344 (41.0)	87 862 (53.4)
Familial disposition for depression	2020 (11.0)	761 (13.3)	11 172 (6.8)
Other major psychiatric disorder	2224 (12.1)	934 (16.3)	2750 (1.7)
BMI^a^			
<18.5	955 (5.2)	353 (6.2)	6996 (4.3)
18.5-24.9	10 135 (55.0)	3184 (55.6)	94 627 (57.5)
25.0-29.9	3806 (20.6)	1143 (20.0)	30 527 (18.6)
≥30.0	2717 (14.7)	798 (13.9)	16 952 (10.3)
Smoker^b^	4352 (23.6)	1515 (26.5)	27 451 (16.7)
Pregestational or gestational diabetes	765 (4.2)	210 (3.7)	4080 (2.5)
Eclampsia or preeclampsia	927 (5.0)	265 (4.6)	7194 (4.4)
Preterm birth^c^	1189 (6.5)	389 (6.8)	10 148 (6.2)
Instrument-assisted delivery	2324 (12.6)	740 (12.9)	22 431 (13.6)
Cesarean delivery	4003 (21.7)	1238 (21.6)	29 470 (17.9)
Medical indication for HC	1413 (7.7)	552 (9.6)	7163 (4.4)
Age at first depression, mean (SD), y	21.5 (3.6)	20.3 (3.4)	NA
Age at exposure-defining episode, mean (SD), y	21.5 (3.6)	21.2 (3.6)	NA
No. of depressive episodes			
0	NA	NA	164 495 (100.0)
1	11 319 (61.4)	2101 (36.7)	NA
2	3832 (20.8)	1359 (23.8)	NA
3	1667 (9.0)	833 (14.6)	NA
≥4	1613 (8.8)	1429 (25.0)	NA

Abbreviations: BMI, body mass index as measured by weight in kilograms divided by height in meters squared; HC, hormonal contraception; NA, not applicable.

^a^ Unknown for 818 (4.4%), 244 (4.3%), and 15 393 (9.4%), for each exposure group, respectively.

^b^ Unknown for 424 (2.3%), 128 (2.2%), and 3981 (2.4%) for each exposure group, respectively.

^c^ Unknown for 102 (0.6%), 34 (0.6%), and 960 (0.6%) for each exposure group, respectively.

**Figure 2.**
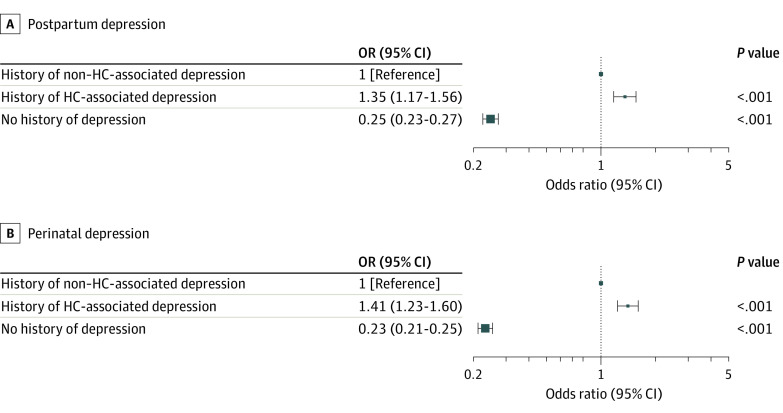
Risk of Postpartum and Perinatal Depression Depending on Depression History Odds ratios (ORs) were adjusted for year of delivery and maternal age, educational level, familial disposition for depression, other major psychiatric disorder, civil status, preterm birth, pregestational or gestational diabetes, eclampsia or preeclampsia, instrument-assisted or cesarean delivery, and medical indication for hormonal contraception (HC) use. HC-associated depression was defined as a depressive episode that developed within 6 months after the start of a new HC exposure. The 95% CIs are plotted on a log scale.

Women with a history of HC-associated depression had a higher risk of PPD than women with a history of non–HC-associated depression, with a crude OR of 1.42 (95% CI, 1.24-1.64) and an adjusted OR of 1.35 (95% CI, 1.17-1.56) (Figure 2A). The risk of PPD was lower for women with no previous depression vs women with non–HC-associated depression, with an adjusted OR of 0.25 (95% CI, 0.23-0.27). A complete summary of results is shown in Table 2. The results remained essentially unchanged in a sensitivity analysis not including obstetric risk factors in the adjustment set (eTable 2 in Supplement 1). When perinatal depression was used as the outcome (ie, including depressive episodes within the third trimester until 6 months post partum), the adjusted OR was 1.41 (95% CI, 1.23-1.60) (Figure 2B; eTable 3 in Supplement 1).

**Table 2.** Risk Factors Associated With Postpartum Depression (PPD)

Risk factor	No. (%)		OR (95% CI)	
	No PPD	PPD	Univariable	Multivariable
Exposure group				
Non–HC-associated depression	17 749 (96.3)	682 (3.7)	1 [Reference]	1 [Reference]
HC-associated depression	5425 (94.8)	297 (5.2)	1.42 (1.24-1.64)	1.35 (1.17-1.56)
No depression	163 017 (99.1)	1478 (0.9)	0.24 (0.22-0.26)	0.25 (0.23-0.27)
Year of delivery				
1996-2001	2598 (99.5)	13 (0.5)	1 [Reference]	1 [Reference]
2001-2006	18 927 (98.8)	226 (1.2)	2.39 (1.42-4.40)	3.03 (1.79-5.62)
2006-2011	58 091 (98.3)	1011 (1.7)	3.48 (2.10-6.35)	4.86 (2.90-8.93)
2011-2016	78 369 (98.8)	940 (1.2)	2.40 (1.45-4.38)	2.97 (1.77-5.48)
2016-June 30, 2017	28 206 (99.1)	267 (0.9)	1.89 (1.13-3.48)	2.34 (1.38-4.35)
Maternal age, y				
12-19	10 256 (97.8)	230 (2.2)	1 [Reference]	1 [Reference]
20-24	56 040 (98.4)	896 (1.6)	0.71 (0.62-0.83)	0.66 (0.56-0.77)
25-29	87 970 (98.9)	951 (1.1)	0.48 (0.42-0.56)	0.55 (0.46-0.65)
30-34	29 595 (98.8)	356 (1.2)	0.54 (0.45-0.63)	0.70 (0.57-0.86)
35-40	2330 (99.0)	24 (1.0)	0.46 (0.29-0.69)	0.57 (0.36-0.88)
Educational level				
Less than high school	43 776 (97.8)	973 (2.2)	1 [Reference]	1 [Reference]
High school or vocational education	75 614 (98.8)	940 (1.2)	0.56 (0.51-0.61)	0.73 (0.66-0.81)
Bachelor’s degree or higher	66 801 (99.2)	544 (0.8)	0.37 (0.33-0.41)	0.57 (0.50-0.65)
Familial disposition for depression				
No	172 516 (98.8)	2179 (1.2)	1 [Reference]	1 [Reference]
Yes	13 675 (98.0)	278 (2.0)	1.61 (1.42-1.82)	1.28 (1.13-1.46)
Other major psychiatric disorder				
No	180 482 (98.8)	2258 (1.2)	1 [Reference]	1 [Reference]
Yes	5709 (96.6)	199 (3.4)	2.79 (2.40-3.22)	1.23 (1.05-1.43)
Married				
No	88 637 (98.4)	1403 (1.6)	1 [Reference]	1 [Reference]
Yes	97 554 (98.9)	1054 (1.1)	0.68 (0.63-0.74)	0.85 (0.78-0.93)
Preterm birth^a^				
No	173 540 (98.7)	2286 (1.3)	1 [Reference]	1 [Reference]
Yes	11 565 (98.6)	161 (1.4)	1.06 (0.90-1.24)	0.96 (0.81-1.13)
Unknown	1086 (99.1)	10 (0.9)	0.70 (0.35-1.23)	0.78 (0.39-1.38)
Pregestational or gestational diabetes				
No	181 233 (98.7)	2360 (1.3)	1 [Reference]	1 [Reference]
Yes	4958 (98.1)	97 (1.9)	1.50 (1.22-1.83)	1.32 (1.07-1.62)
Eclampsia or preeclampsia				
No	177 948 (98.7)	2314 (1.3)	1 [Reference]	1 [Reference]
Yes	8243 (98.3)	143 (1.7)	1.33 (1.12-1.58)	1.28 (1.07-1.51)
Instrument-assisted or cesarean delivery				
No	129 004 (98.8)	1611 (1.2)	1 [Reference]	1 [Reference]
Yes	57 187 (98.5)	846 (1.5)	1.18 (1.09-1.29)	1.16 (1.07-1.27)
Medical indication for HC use				
No	177 223 (98.7)	2297 (1.3)	1 [Reference]	1 [Reference]
Yes	8968 (98.2)	160 (1.8)	1.38 (1.17-1.61)	1.18 (1.00-1.39)

Abbreviations: HC, hormonal contraception; OR, odds ratio.

^a^ Missing for 0.6% for each exposure group, which was handled by grouping women with missing data in a separate group.

To exclude a potential contribution of HC use post partum to PPD incidence, women who started using HC after delivery but before they developed PPD were excluded in a sensitivity analysis. The proportions of women starting an HC post partum were 40.8% vs 42.0% in those with a history of non–HC-associated vs HC-associated depression. The adjusted OR was 1.44 (95% CI, 1.23-1.69) (eTable 4 in Supplement 1).

When the 90-day and 180-day treatment-free intervals were used to distinguish depressive episodes, the proportions of women with more than 1 depressive episode were 27.6% vs 49.7% and 22.1% vs 41.3% in those with prior non–HC-associated vs HC-associated depression, respectively. The adjusted ORs between PPD and HC-associated depression were 1.33 (95% CI, 1.14-1.53) and 1.32 (95% CI, 1.14-1.53) (eTable 5 in Supplement 1).

When exposure was classified based on women’s first depressive episode, 3792 (2.0%) and 20 361 (10.8%) women were classified as having a history of HC-associated vs non–HC-associated depression. The proportions of women who had more than 1 depressive episode were similar between the groups (44.4% vs 44.6% of women with non–HC-associated vs HC-associated depression) (eTable 6 in Supplement 1). The adjusted OR for developing PPD was 1.19 (95% CI, 1.00-1.40) and for perinatal depression, 1.18 (95% CI, 1.01-1.38). After excluding women who started HC post partum but before PPD onset, the adjusted OR was 1.24 (95% CI, 1.02-1.49) (eTable 7 in Supplement 1).

## Discussion

This population-based cohort study of 188 648 first-time mothers provides evidence for the existence of a subgroup of women who are sensitive to hormonal transitions across their reproductive lives by showing an association between 2 types of depressive episodes with plausible hormonal contributions. The findings show that women with a history of depression associated with HC initiation had a higher risk of developing a depressive episode during pregnancy and after childbirth compared with women with a history of depression not associated with HC initiation.

Our findings contribute new evidence for an association between depressive episodes across hormonal transitions in the reproductive life span, supporting the existence of a hormone-sensitive subgroup of women.^8^ Our findings also align with previous findings suggesting an association between PPD and the retrospective reporting of experienced mood deterioration associated with HC use.^13,14^ Furthermore, the finding of a similar association with perinatal depression, ie, when both late pregnancy and postpartum onset of depressive episodes were included, suggests that both the pregnancy (ie, during high hormone levels) and postnatal (ie, during abrupt hormone decline) states contribute to the mechanisms by which depression emerges in women who may be sensitive to HC exposure. This outcome is in line with findings from a study that compared women with and without a history of PPD who underwent pharmacologic sex hormone manipulation; women with a history of PPD developed depressive symptoms both during the withdrawal phase of a pharmacologic hormone manipulation and the subsequent hormone add back.^25^

The mechanistic understanding of how changes in the hormone milieu induce depressive symptoms in some women, but not in others, is far from well established. Some evidence points toward a genetic predisposition; for example, a large twin study of the psychiatric adverse effects of oral contraceptives found a distinct genetic basis for depressive symptoms associated vs not associated with HC use.^26^ Furthermore, a specific pattern of gene expression during pregnancy shows a high level of accuracy in predicting the development of PPD, and many of these genes are suggested to be involved in estrogen receptor signaling.^27^ Notably, this finding translates to a pharmacologic sex hormone manipulation study in healthy women where the pharmacologically induced change in a subset of these gene transcripts correlated with the emergence of depressive symptoms and changes in a marker of brain serotonin signaling.^28^ This finding indicates that serotonin-related brain mechanisms may be involved in the pathophysiology of hormone-triggered depressive symptoms.^29^ Furthermore, hormones, including HC, may affect the monoaminergic brain system, especially the serotonin system, which may play a key role in reproductive mood disorders.^2,30,31,32,33^

This work contributes evidence to guide clinical PPD risk stratification and potentially improve PPD prediction models.^34^ Future work should evaluate risk models for PPD that include information on previous depressive episodes and subclinical depressive symptoms associated with HC use, which could potentially further inform a stratified approach to reproductive care. These risk models could be a useful tool in future precision medicine, as some women may benefit more from prophylactic strategies or treatments targeting the hormonal mechanisms of depression.^35,36^ Furthermore, future work should investigate whether our findings can be generalized to depressive episodes associated with other hormonal transitions, such as depression in perimenopause.

### Strengths and Limitations

The strengths of the study include the use of national registers to obtain health data on a large population over 23 years. The use of registry data enabled us to obtain extensive health information on all women living in Denmark from when they were maximally 16 years of age without the risk of recall bias.

The study also has some limitations. First, our study is based on the assumption that HC use is associated with an increased risk of depression at the population level. However, at the individual level we were not able to verify whether a depressive episode developed because of HC use. In addition, we were not able to detect women who developed depression while using HC but were not treated with antidepressants or diagnosed with depression at a psychiatric inpatient or outpatient clinic; hence, the magnitude of the associated PPD risk should be interpreted with caution. Furthermore, by using prescription of antidepressants or depression diagnosis to measure depressive episodes, we may have only captured the most severe cases. We acknowledge that our findings may not necessarily be generalizable to mild depressive episodes. Second, the use of antidepressant prescriptions as a proxy for depression can introduce misclassification bias, as antidepressants are used for other indications, such as anxiety and obsessive-compulsive disorder. In Denmark, however, 60% to 80% of prescribed antidepressants are used for treating depression.^37,38^ Third, using our defined time gap between treatments to define new onsets of depressive episodes might not always apply, as time gaps in treatment can be due to periods of noncompliance or a mismatch between the daily dose used and the defined daily dose. This potential misclassification of new-onset depressive episodes is reflected by the large reduction in the number of depressive episodes that was observed when a 90-day and a 180-day grace period were used instead of a 30-day grace period between treatments. However, the results from the sensitivity analyses with longer grace periods did not differ markedly from the main analysis. Fourth, women with HC-associated depression had more depressive episodes than women with non–HC-associated depression, perhaps because prior depression has been shown to be associated with a higher risk of subsequent recurrent depression triggered by HC use.^39^ However, if due to other reasons, then the higher number of depressive episodes may increase the likelihood that an episode will coincide with a new HC exposure by chance. This coincidence may induce a bias, as we expect that a history of recurrent depressive episodes compared with a single episode is associated with a higher risk of PPD. Nonetheless, this does not explain the observed association between HC-related depression and PPD, because when the exposure groups were defined based on the first depressive episode, the groups showed similar numbers of depressive episodes and the risk of PPD was, though less pronounced, still higher in the women with HC-associated depression compared with those with non–HC-associated depression. Fifth, a potential influence of unmeasured confounders cannot be excluded, such as differences in prescription patterns; however, by only including ever-users of HC and by comparing groups of women with a history of depression, the risk of confounding was minimized. Furthermore, no diagnosis code exists for PMDD in the 8th and 10th revisions of the *International Classification of Diseases and Related Health Problems*, which could be a potential confounder. Such confounding would, however, still provide evidence for hormonal sensitivity being associated with an increased risk of depressive episodes across the reproductive life span in a subgroup of women.

## Conclusions

This study provides evidence for the existence of a subgroup of women who are sensitive to hormonal transitions across the reproductive life span by showing that a history of depression coinciding with the initiation of HC may be associated with a higher risk of PPD beyond the risk of a history of depression not coinciding with HC initiation. Importantly, the findings do not imply that HC use leads to a higher risk of PPD but do indicate that a history of HC-associated depression may unmask PPD susceptibility, which may prove useful as a clinical tool in PPD risk stratification.

## Notes

Supplement 1. **eTable 1.** Overview of Registers, Variables, and Codes **eTable 2.** Without Adjusting for Obstetric Risk Factors for Postpartum Depression **eTable 3.** History of Hormonal Contraceptive-Associated Depression and Risk of Perinatal Depression **eTable 4.** Without Women With Postpartum Depression Potentially Triggered by Hormonal Contraceptive Use **eTable 5.** Depressive Episodes Are Distinguished by a Minimum of 90-Day and 180-Day Treatment-Free Intervals **eTable 6.** Demographic Characteristics and Clinical Profiles When Exposure Is Defined Based on First Depression **eTable 7.** Exposure Defined at First Depressive Episode

Supplement 2.
Data Sharing Statement
