# Supplementary material for: Depression Associated With Hormonal Contraceptive Use as a Risk Indicator for Postpartum Depression
Source: JAMA Psychiatry. 2023 Apr 26;80(7):682–9. doi: 10.1001/jamapsychiatry.2023.0807 (PMC10134043; doi:10.1001/jamapsychiatry.2023.0807)
Supplement: Supplement 1. — eTable 1. Overview of Registers, Variables, and Codes eTable 2. Without Adjusting for Obstetric Risk Factors for Postpartum Depression eTable 3. History of Hormonal Contraceptive-Associated Depression and Risk of Perinatal Depression eTable 4. Without Women With Postpartum Depression Potentially Triggered by Hormonal Contraceptive Use eTable 5. Depressive Episodes Are Distinguished by a Minimum of 90-Day and 180-Day Treatment-Free Intervals eTable 6. Demographic Characteristics and Clinical Profiles When Exposure Is Defined Based on First Depression eTable 7. Exposure Defined at First Depressive Episode [file jamapsychiatry-e230807-s001.pdf]

Supplemental Online Content

Larsen SV, Mikkelsen AP, Lidegaard O, Frokjaer VG. Depression associated with hormonal contraceptive use as a risk indicator for postpartum depression. *JAMA Psychiatr*. Published online April 26, 2023. doi:10.1001/jamapsychiatry.2023.0807

eTable 1. Overview of Registers, Variables, and Codes

eTable 2. Without Adjusting for Obstetric Risk Factors for Postpartum Depression

eTable 3. History of Hormonal Contraceptive-Associated Depression and Risk of Perinatal Depression

eTable 4. Without Women With Postpartum Depression Potentially Triggered by Hormonal Contraceptive Use

eTable 5. Depressive Episodes Are Distinguished by a Minimum of 90-Day and 180-Day Treatment-Free Intervals

eTable 6. Demographic Characteristics and Clinical Profiles When Exposure Is Defined Based on First Depression

eTable 7. Exposure Defined at First Depressive Episode

This supplemental material has been provided by the authors to give readers additional information about their work.

© 2023 Larsen SV et al. *JAMA Psychiatry*.

eTable 1. Overview of Registers, Variables, and Codes

Registers	Variables	Codes
**Danish Civil Registration System** (data since 1968)		
	Date of birth, immigration, emigration, kinship, civil status	
**Demographic Registers of Statistics Denmark**		
	Educational degree	
**Danish Medical Birth Register** (complete data since 1973)		
	Twin birth, stillbirth, gestational age, BMI, smoking status	
**Danish National Patient Register** (complete data since 1977)		
	Postpartum/perinatal depression	ICD-8: 296.09, 296.29, 298.09, 300.49, 301.19<br>ICD-10: DF32-34, DF38, DF39, DF530
	Instrument-assisted and caesarean delivery	ICD-10: DO81, DO82<br>NCSP-D: KMAE, KMAF0-2, KMAG03, KMAG13, KMCA
	Preeclampsia/eclampsia	ICD-8: 637, ICD-10: DO11, DO14-15
	Pre-gestational/gestational diabetes	ICD-8: 249-250<br>ICD-10: DE10-14, DO24
Medical indications for HC use	Polycystic ovary syndrome	ICD-8: 256.9<br>ICD-10: DE282
	Endometriosis	ICD-8: 625.3<br>ICD-10: DN80
	Premenstrual syndrome	ICD-10: DN943
	Dysmenorrhea	ICD-8: 626.3<br>ICD-10: DN944-946
	Heavy menstrual bleeding	ICD-8: 626.2<br>ICD-10: DN92
	Hirsutism	ICD-10: DL680
	Acne	ICD-8: 706.1<br>ICD-10: DL70
**The Psychiatric Central Register** (complete data since 1969 on hospital admission and 1995 on outpatient contacts)		
	Depression diagnosis	ICD-8: 296.09, 296.29, 298.09, 300.49, 301.19<br>ICD-10: DF32-34, DF38, DF39
Other major psychiatric disorders	Organic, including symptomatic, mental disorders	ICD-8: 290.09-11, 290.18-9, 292-294<br>ICD-10: DF00-09
	Mental and behavioral disorders due to psychoactive substance use	ICD-8: 291, 303.09, 303.19-20, 303.28-9, 303.91, 303.99, 304<br>ICD-10: DF10-DF19, DF55
	Schizophrenia, Schizotypal and delusional disorders	ICD-8: 295, 297-9<br>ICD-10: DF20-DF29
	Mood disorders	ICD-8: 296.19, 296.39, 296.89, 296.99<br>ICD-10: DF30-31
	Eating Disorder	ICD-8: 306.5<br>ICD-10: DF50
	Mental retardation	ICD-8: 310-4<br>ICD-10: DF7
**Danish Prescription Register** (complete data since 1995)		
	Hormonal contraception	ATC: Codes starting with G03A (except G03AD), G02BA03, G02BB01, and G03HB01
	Antidepressant medication	ATC: Codes starting with N06A

ATC: Anatomical Therapeutic Chemical Classification system; ICD-8: International Classification of Disease and Health Related Problems, 8th revision; ICD-10: 10th revision, NCSP-D: Nordic Medico-Statistical Committee (NOMESCO) Classification of Surgical Procedures – Denmark.

© 2023 Larsen SV et al. *JAMA Psychiatry*.

eTable 2. Without Adjusting for Obstetric Risk Factors for Postpartum Depression

Risk factors		No PPD (%)	PPD (%)	OR (95% CI) (univariable)	OR (95% CI) (multivariable)
Exposure group	Non–HC-associated depression	17,749 (96.3)	682 (3.7)	Ref	Ref
	HC-associated depression	5,425 (94.8)	297 (5.2)	1.42 (1.24-1.64)	1.35 (1.17-1.55)
	No depression	163,017 (99.1)	1,478 (0.9)	0.24 (0.22-0.26)	0.25 (0.22-0.27)
Year of delivery	(1996, 2001]	2,598 (99.5)	13 (0.5)	Ref	Ref
	(2001, 2006]	18,927 (98.8)	226 (1.2)	2.39 (1.42-4.40)	3.05 (1.80-5.65)
	(2006, 2011]	58,091 (98.3)	1,011 (1.7)	3.48 (2.10-6.35)	4.91 (2.93-9.02)
	(2011, 2016]	78,369 (98.8)	940 (1.2)	2.40 (1.45-4.38)	3.01 (1.79-5.54)
	(2016, June 2017]	28,206 (99.1)	267 (0.9)	1.89 (1.13-3.48)	2.36 (1.39-4.39)
Maternal age	(12,20]	10,256 (97.8)	230 (2.2)	Ref	Ref
	(20,25]	56,040 (98.4)	896 (1.6)	0.71 (0.62-0.83)	0.67 (0.57-0.78)
	(25,30]	87,970 (98.9)	951 (1.1)	0.48 (0.42-0.56)	0.56 (0.47-0.67)
	(30,35]	29,595 (98.8)	356 (1.2)	0.54 (0.45-0.63)	0.73 (0.60-0.89)
	(35,40]	2,330 (99.0)	24 (1.0)	0.46 (0.29-0.69)	0.61 (0.38-0.94)
Educational level	<high school	43,776 (97.8)	973 (2.2)	Ref	Ref
	High school/vocational	75,614 (98.8)	940 (1.2)	0.56 (0.51-0.61)	0.73 (0.66-0.81)
	≥Bachelor degree	66,801 (99.2)	544 (0.8)	0.37 (0.33-0.41)	0.56 (0.49-0.64)
Familial disposition for depression	No	172,516 (98.8)	2,179 (1.2)	Ref	Ref
	Yes	13,675 (98.0)	278 (2.0)	1.61 (1.42-1.82)	1.28 (1.13-1.46)
Other major psychiatric disorder	No	180,482 (98.8)	2,258 (1.2)	Ref	Ref
	Yes	5,709 (96.6)	199 (3.4)	2.79 (2.40-3.22)	1.22 (1.04-1.42)
Married	No	88,637 (98.4)	1,403 (1.6)	Ref	Ref
	Yes	97,554 (98.9)	1,054 (1.1)	0.68 (0.63-0.74)	0.85 (0.78-0.93)
Medical indication for HC use	No	177,223 (98.7)	2,297 (1.3)	Ref	Ref
	Yes	8,968 (98.2)	160 (1.8)	1.38 (1.17-1.61)	1.20 (1.01-1.41)

PPD, postpartum depression. HC: hormonal contraceptive.

© 2023 Larsen SV et al. *JAMA Psychiatry*.

eTable 3. History of Hormonal Contraceptive-Associated Depression and Risk of Perinatal Depression

History of depression	No PPD (%)	PPD (%)	OR (95% CI) (univariable)	OR (95% CI) (multivariable)
Non–HC-associated depression	17,749 (95.8)	787 (4.2)	Ref	Ref
HC-associated depression	5,425 (93.8)	356 (6.2)	1.48 (1.30-1.68)	1.41 (1.23-1.60)
No depression	163,017 (99.0)	1,579 (1.0)	0.22 (0.20-0.24)	0.23 (0.21-0.25)

PPD, postpartum depression. HC: hormonal contraceptive.

© 2023 Larsen SV et al. *JAMA Psychiatry*.

eTable 4. Without Women With Postpartum Depression Potentially Triggered by Hormonal Contraceptive Use

History of depression	No PPD (%)	PPD (%)	OR (95% CI) (univariable)	OR (95% CI) (multivariable)
Non–HC-associated depression	17,749 (97.3)	493 (2.7)	Ref	Ref
HC-associated depression	5,425 (96.0)	228 (4.0)	1.51 (1.29-1.77)	1.44 (1.23-1.69)
No depression	163,017 (99.3)	1,073 (0.7)	0.24 (0.21-0.26)	0.25 (0.22-0.28)

PPD, postpartum depression. HC: hormonal contraceptive.

© 2023 Larsen SV et al. *JAMA Psychiatry*.

eTable 5. Depressive Episodes Are Distinguished by a Minimum of 90-day and 180-day Treatment-Free Intervals

90-days treatment free interval

	Exposure	
	**Non–HC-associated depression**	**HC-associated depression**
Number of depressive episodes, no. (%)		
1	13,826 (72.4)	2,546 (50.3)
2	3,628 (19.0)	1,396 (27.6)
3	1,118 (5.9)	642 (12.7)
≥4	516 (2.7)	481 (9.5)

History of depression	No PPD (%)	PPD (%)	OR (95% CI) (univariable)	OR (95% CI) (multivariable)
Non–HC-associated depression	18,372 (96.2)	716 (3.8)	Ref	Ref
HC-associated depression	4,802 (94.8)	263 (5.2)	1.41 (1.21-1.62)	1.33 (1.14-1.53)
No depression	163,017 (99.1)	1,478 (0.9)	0.23 (0.21-0.25)	0.24 (0.22-0.27)

180-days treatment free interval

	Exposure	
	**Non–HC-associated depression**	**HC-associated depression**
Number of depressive episodes, no. (%)		
1	15,102 (77.9)	2,794 (58.7)
2	3,313 (17.1)	1,306 (27.4)
3	756 (3.9)	450 (9.5)
≥4	221 (1.1)	211 (4.4)

History of depression	No PPD (%)	PPD (%)	OR (95% CI) (univariable)	OR (95% CI) (multivariable)
Non–HC-associated depression	18,661 (96.2)	731 (3.8)	Ref	Ref
HC-associated depression	4,513 (94.8)	248 (5.2)	1.40 (1.21-1.62)	1.32 (1.14-1.53)
No depression	163,017 (99.1)	1,478 (0.9)	0.23 (0.21-0.25)	0.24 (0.22-0.27)

PPD, postpartum depression. HC, hormonal contraceptive.

© 2023 Larsen SV et al. *JAMA Psychiatry*.

eTable 6. Demographic Characteristics and Clinical Profiles When Exposure Is Defined Based on First Depression

Profiles	Exposure, No. (%)		
	Non–HC-associated depression	HC-associated depression	No depression
Total	20,361 (10.8)	3,792 (2.0)	164,495 (87.2)
Maternal age at delivery, y			
<20	456 (2.2)	150 (4.0)	9,880 (6.0)
20-24	5,743 (28.2)	1,308 (34.5)	49,885 (30.3)
25-29	9,535 (46.8)	1,648 (43.5)	77,738 (47.3)
30-34	4,151 (20.4)	629 (16.6)	25,171 (15.3)
35-39	476 (2.3)	57 (1.5)	1,821 (1.1)
Educational level			
Less than high school	6,253 (30.7)	1,461 (38.5)	37,035 (22.5)
High school or vocational education	8,473 (41.6)	1,436 (37.9)	66,645 (40.5)
Bachelor's degree or higher	5,635 (27.7)	895 (23.6)	60,815 (37.0)
Married	9,185 (45.1)	1,561 (41.2)	87,862 (53.4)
Familial disposition for depression	2,300 (11.3)	481 (12.7)	11,172 (6.8)
Other major psychiatric disorder	2,637 (13.0)	521 (13.7)	2,750 (1.7)
BMI[a]			
<18.5	1,076 (5.3)	232 (6.1)	6,996 (4.3)
18.5 - 24.9	11,210 (55.1)	2,109 (55.6)	94,627 (57.5)
25.0-29.9	4,206 (20.7)	743 (19.6)	30,527 (18.6)
≥30.0	2,987 (14.7)	528 (13.9)	16,952 (10.3)
Smoker[b]	4,845 (23.8)	1,022 (27.0)	27,451 (16.7)
Pregestational or gestational diabetes	846 (4.2)	129 (3.4)	4,080 (2.5)
Eclampsia or preeclampsia	1,018 (5.0)	174 (4.6)	7,194 (4.4)
Preterm birth[c]	1,312 (6.4)	266 (7.0)	10,148 (6.2)
Instrument-assisted delivery	2,597 (12.8)	467 (12.3)	22,431 (13.6)
Cesarean delivery	4,437 (21.8)	804 (21.2)	29,470 (17.9)
Medical indication for HC	1,615 (7.9)	350 (9.2)	7,163 (4.4)
Age at first depression, mean (SD), y	21.3 (3.6)	20.6 (3.4)	-
No. of depressive episodes			
0	-	-	164,495 (100.0)
1	11,319 (55.6)	2,101 (55.4)	-
2	4,374 (21.5)	817 (21.5)	-
3	2,113 (10.4)	387 (10.2)	-
≥4	2,555 (12.5)	487 (12.8)	-

[a]882 (4.3%) of the first-time mothers with a history of non-HC-triggered depression, 180 (4.7%) of those with HC-triggered depression, and 15,393 (9.4%) of those with no history of depression had unknown BMI. [b]Correspondingly, 474 (2.3%), 78 (2.1%), and 3,981 (2.4%) had unknown smoking status, and [c]111 (0.5%), 25 (0.7%) and 960 (0.6%) had unknown gestational age from each exposure group, respectively.

BMI, Body Mass Index as measured by weight in kilograms divided by height in meters squared; HC, hormonal contraceptive.

© 2023 Larsen SV et al. *JAMA Psychiatry*.

eTable 7. Exposure Defined at First Depressive Episode

History of depression	No PPD (%)	PPD (%)	OR (95% CI) (univariable)	OR (95% CI) (multivariable)
**Outcome: Postpartum depression**				
Non–HC-associated	19,567 (96.1)	794 (3.9)	Ref	Ref
HC-associated	3,607 (95.1)	185 (4.9)	1.26 (1.07-1.49)	1.19 (1.00-1.40)
No depression	163,017 (99.1)	1,478 (0.9)	0.22 (0.20-0.24)	0.24 (0.22-0.26)
**Outcome: Perinatal depression**				
Non–HC-associated	19,567 (95.5)	928 (4.5)	Ref	Ref
HC-associated	3,607 (94.4)	215 (5.6)	1.26 (1.08-1.46)	1.18 (1.01-1.38)
No depression	163,017 (99.0)	1,579 (1.0)	0.20 (0.19-0.22)	0.22 (0.20-0.24)
**Outcome: Postpartum depression not potentially triggered by hormonal contraceptive use**				
Non–HC-associated	19,567 (97.1)	581 (2.9)	Ref	Ref
HC-associated	3,607 (96.3)	140 (3.7)	1.31 (1.08-1.57)	1.24 (1.02-1.49)
No depression	163,017 (99.3)	1,073 (0.7)	0.22 (0.20-0.25)	0.24 (0.21-0.26)

PPD, postpartum depression. HC, hormonal contraceptive.

© 2023 Larsen SV et al. *JAMA Psychiatry*.
