# Supplementary material for: Depression Associated With Hormonal Contraceptive Use as a Risk Indicator for Postpartum Depression
Source: JAMA Psychiatry. 2023 Apr 26;80(7):682–9. doi: 10.1001/jamapsychiatry.2023.0807 (PMC10134043; doi:10.1001/jamapsychiatry.2023.0807)
Supplement: Supplement 2. — Data Sharing Statement [file jamapsychiatry-e230807-s002.pdf]

Data Sharing Statement

Data
Data available: No

Additional Information
Explanation for why data not available: Danish national health register data cannot be distributed, but access to the data can be granted by the appropriate authorities.
